# Complete mitochondrial genome of *Amorophaga japonica* Robinson, 1986 (Lepidoptera: Tineidae)

**DOI:** 10.1080/23802359.2020.1774437

**Published:** 2020-06-05

**Authors:** Jong Seok Kim, Min Jee Kim, Sung Soo Kim, Iksoo Kim

**Affiliations:** a Department of Applied Biology, College of Agriculture & Life Sciences, Chonnam National University, Gwangju, Republic of Korea; b Herbal Medicine Resources Research Center, Korea Institute of Oriental Medicine, Naju, Republic of Korea; c Research Institute for East Asian Environment and Biology, Seoul, Republic of Korea

**Keywords:** Mitochondrial genome, Tineidae, *Amorophaga japonica*, phylogeny

## Abstract

The complete mitochondrial genome (mitogenome) of *Amorophaga japonica* Robinson, 1986 (Lepidoptera: Tineidae), comprises 15,027 base pairs (bp) and contains a typical set of genes (13 protein-coding genes [PCGs], 2 rRNA genes, and 22 tRNA genes), and 1 non-coding region. The genome has an arrangement, *trnW*-*trnY*-*trnC*, instead of typical *trnW*- *trnC*-*trnY* at the *ND2* and *COI* junction. This arrangement is unique in lepidopteran mitogenomes. Unlike most lepidopteran insects, which have CGA as the start codon for the *COI* gene sequence, *A. japonica COI* had a typical ATT codon. The A + T-rich region was unusually short, with only 199 bp. Phylogenetic analyses with concatenated sequences of the 13 PCGs and two rRNA genes using the Bayesian inference method placed *A. japonica* in Tineidae as a sister to the cofamilial species, *Tineola bisselliella*, with high nodal support (Bayesian posterior probability [BPP] = 0.99), presenting the superfamily Tineoidea in a monophyletic group with a BPP of 0.99. Gracillarioidea, represented by three species of Gracillariidae, formed a monophyletic group with the highest BPP, but the *Leucoptera malifoliella* in Yponomeutoidea was unusually grouped together with the Gracillarioidea with the highest nodal support. As more mitogenome sequences are available, further analysis to infer the relationships among superfamilies of Lepidoptera might be possible.

Four species of *Amorophaga* are recognized globally, and *A. japonica* is distributed throughout China, Korea, and Japan (Zagulajev 1975; Robinson 1986). This species feeds on fruit bodies of the wood-decaying bracket fungus *Cryptoporus volvatus* (Polyporaceae: Basidiomycota) in Japan (Kadowaki and Yamazoe 2011). The larvae of the species pupate in interstices of fruit bodies (Osada et al. 2015).

An adult male *A. japonica* was collected from Naechon-myeon, Pocheon City, Gyeonggido Province, South Korea (37°48′37.0ʺN, 127°15′21.7ʺE) in 2013. This voucher specimen was deposited at the Chonnam National University, Gwangju, Korea, under Accession No. CNU7296. Using DNA extracted from the hind legs, three long overlapping fragments (LFs; *COI*-*ND4*, *ND5*-*lrRNA*, and *lrRNA*-*COI*) were amplified using previously described primers (Kim et al. 2012). These three LFs were used as templates to amplify 26 short fragments (Kim et al. 2012).

Phylogenetic analysis using the concatenated nucleotide sequences of 13 protein-coding genes (PCGs) and two rRNA genes was performed using the Bayesian inference (BI) method implemented in CIPRES Portal v. 3.1 (Miller et al. 2010). An optimal partitioning scheme (six partitions) and substitution model (GTR + Gamma + I) were determined using PartitionFinder 2 and the greedy algorithm (Lanfear et al. 2012, 2014, 2016).

The complete 15,027-base pair (bp) mitochondrial genome (mitogenome) of *A. japonica* was composed of typical gene sets (2 rRNAs, 22 tRNAs, and 13 PCGs) and a major non-coding A + T-rich region (GenBank accession no. MH823253). The length of the *A. japonica* A + T-rich region was the second shortest (199 bp), next to *Eumeta variegata* (94 bp; Jeong et al. 2018), among sequenced Tineoidea, Gracillarioidea, and Yponomeutoidea (94–1610 bp; data not shown). The genome has an arrangement *trnW*-*trnY*-*trnC*, instead of typical *trnW*-*trnC*-*trnY* at the *ND2* and *COI* junction, presenting a new gene arrangement in lepidopteran mitogenomes (Park et al. 2016). All PCGs contained the typical ATN start codon, including *COI* (four ATT, three ATA, and six ATG). This differs from the start codon for *COI* in other available species of Tineoidea, Gracillarioidea, and Yponomeutoidea (data not shown), as well as most species of Lepidoptera (Kim et al. 2010). The A/T content of the whole mitogenome was 81.6%; however, it varied among the genes as follows: A + T-rich region, 95.5%; *srRNA*, 86.9%; *lrRNA*, 86.5%; tRNAs, 83.2%; and PCGs, 80.1%.

Phylogenetic analyses performed using the concatenated sequences of the 13 PCGs and 2 rRNA genes using the BI method placed *A. japonica* in Tineidae as the sister to a cofamilial species, *Tineola bisselliella*, with high nodal support (Bayesian posterior probability [BPP] = 0.99) (Figure 1). It also placed *E. variegata* and *Mahasena oolona*, which belong to the family Psychidae as sister species to each other, presenting Tineoidea as a monophyletic group with high nodal support (BPP = 0.98). Gracillarioidea, which was represented by three species of Gracillariidae formed a monophyletic group with the highest BPP, but *Leucoptera malifoliella,* belonging to the family Lyonetiidae in Yponomeutoidea, was unusually grouped together with Gracillarioidea with the highest nodal support (BPP = 1). Currently, only limited mitogenome sequences are available from Gracillarioidea, Tineoidea, and Yponomeutoidea. Therefore, atypical relationships are unavoidable. Nevertheless, a previous large-scale phylogenetic analyses using 14,658 characters from 19 nuclear PCGs for lepidopteran phylogeny instead has shown a closer relationship of the genus *Leucoptera* in Lyonetiidae to Yponomeutoidea (Regier et al. 2013). Therefore, an additional, scrutinized analysis with extended taxon sampling is essential to verify current unexpected relationships.

**Figure 1.**
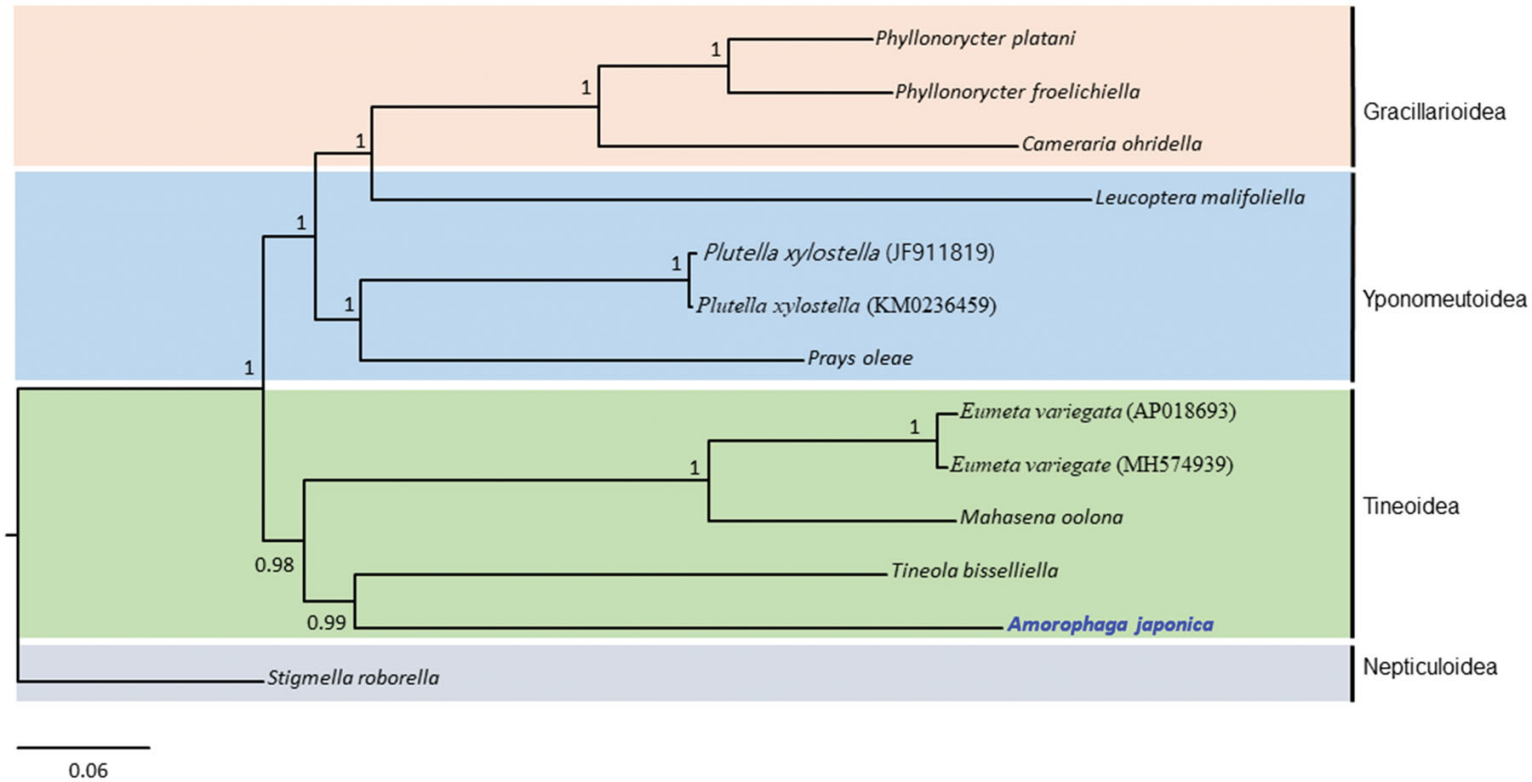
Bayesian inference (BI) method-based phylogenetic tree for three superfamilies in Ditrysia (Tineoidea, Gracillarioidea, and Yponomeutoidea) obtained using concatenated sequences of 13 PCGs and 2 rRNAs. The numbers at each node indicate Bayesian posterior probabilities (BPPs). The scale bar indicates the number of substitutions per site. One species of Nepticuloidea (*Stigmella roborella*) was included as an outgroup. GenBank accession numbers are as follows: *Tineola bisselliella*, KJ508045 (Timmermans et al. 2014); *Mahasena oolona*, KY856825 (Li et al. 2017); *Eumeta variegate*, AP018693 and MH574939 (Arakawa et al. 2018; Jeong et al. 2018); *Phyllonorycter froelichiella*, KJ508048 (Timmermans et al. 2014); *Phyllonorycter platani*, KJ508044 (Timmermans et al. 2014); *Cameraria ohridella*, KJ508042 (Timmermans et al. 2014); *Plutella xylostella*, JF911819 and KM023645 (Wei et al. 2013; Dai et al. 2016); *Leucoptera malifoliella*, JN790955 (Wu et al. 2012); *Prays oleae*, KM874804 (van Asch et al. 2014); and *Stigmella roborella*, KJ508054 (Timmermans et al. 2014).

## Data Availability

The data that support the findings of this study are openly available in Mendeley Data at http://dx.doi.org/10.17632/yhmx6hpysr.1.
